# School-based interventions to promote personal and environmental hygiene practices among children in Pakistan: protocol for a mixed methods study

**DOI:** 10.1186/s12889-020-08511-0

**Published:** 2020-04-14

**Authors:** Nousheen Akber Pradhan, Waliyah Mughis, Tazeen Saeed Ali, Maleeha Naseem, Rozina Karmaliani

**Affiliations:** 1 Department of Community Health Sciences (CHS), Aga Khan University (AKU), Karachi, Pakistan; 2 Department of Pediatrics & Child Health, Aga Khan University Hospital, Karachi, Pakistan; 3 School of Nursing & Midwifery, Aga Khan University, Karachi, Pakistan

**Keywords:** School-based interventions, Hygiene practices, Pakistan, Hygiene interventions, School children

## Abstract

**Background:**

Poor personal hygiene and inadequate sanitation practices among young children leads to communicable diseases. There remains a gap in the holistic assessment of school children’s hygiene literacy, practices and effectiveness of school-based hygiene interventions in Pakistan. Therefore, a school-based intervention protocol has been designed to promote personal and environmental hygiene practices for primary school children. Towards improving children’s hygiene behaviors, the study will also focus on enhancing mothers' hygiene knowledge and practices.

**Methods:**

Using quasi-experimental design with mixed methods data collection approaches, this study will be conducted in schools located in an urban squatter settlements in Pakistan. To assess primary grade children and their mothers‘ hygiene status, a survey will be held in the pre-intervention phase. This phase also includes qualitative exploration of key stakeholders (mothers, teachers, health & education authorities representatives’) perceptions about the factors facilitating and impeding the adaption of hygiene behaviors among school children. In-depth guides and focus group discussion tools will be used for this purpose. This will be followed by multi-component intervention phase with behavior change strategies to improve children‘s and mothers’ hygiene literacy and behaviors. The post-intervention phase will assess the intervention effectiveness in terms of enhancing hygiene knowledge and practices among school children and mothers, alongside exploration of mothers and teachers’ insights into whether or not the intervention has been effective in improving hygiene practices among children. Paired *t*-test will be applied pre and post-intervention to measure the differences between the mothers and children's hygiene literacy and knowledge scores. Similar test will be performed to assess the differences in children’s hygiene knowledge and practice scores, pre and post-intervention (< 50 = poor, 50–75 = good and > 75 = excellent). Thematic analysis will be carried out for qualitative data.

**Discussion:**

Multi-component intervention aimed at improving personal and environmental hygiene among primary school children offers an opportunity to design and test various behavioral change strategies at school and in home settings. The study findings will be significant in assessing the intervention’s effectiveness in improving children‘s overall hygiene.

**Trial registration:**

Retrospectively registered with ClinicalTrials.gov (NCT03942523) on 5th May 2019.

## Background

Globally, communicable diseases are prevalent among school age children and exposure to variety of pathogens causing preventable diseases in school population is inevitable. Underlying factors mainly rest on poor personal hygiene and inadequate sanitation practices [1], resulting in school absenteeism which affects academic performance of children due to illness [2]. The situation is worse in low and middle income countries (LMICs) due to inadequate health care facilities, leading to compromised health status of school children [3–5].

Diarrhea and respiratory illnesses; communicable diseases associated with poor hygiene are regarded as the deadliest killers of young children [6]. Incidence of diarrheal diseases in the initial years has been linked with impaired cognitive performance in the later childhood [7, 8]. In developing countries, intestinal helminthic infection is a commonly cited problem among school age children [9–11]. Furthermore, oral health infections are also commonly found in school going children worldwide [4]. Frequent attacks of infection predispose young children to malnutrition. This can lead to vicious cycle and retard children's physical and cognitive development [12].

School children’s hygiene literacy and practices have therefore received considerable attention to control the spread of infections among this group [13]. Infections due to poor knowledge and unhygienic habits of young children lead to compromised academic performance [12]. Knowledge, Attitude, and Practice (KAP) survey of primary school students in Ethiopia indicated that almost half of the students had adequate knowledge of hygiene. However, the practice of handwashing with soap was not appreciable (36%) [1]. A survey in Palestine showed that 68% of the students reported washing hands with soap after using toilets, playing, and eating [14]. A study in India demonstrated that majority of the students’ correct knowledge about handwashing before meals, brushing teeth, rinsing mouth after eating, and combing hairs; did not translate into correct practice in all the cases [12], indicating the significance of behavior change reinforcement strategies.

Moreover, the contextual factors such as poor socio-economic environment further deteriorates the health status of school children, especially in LMICs. The physical environment of school also plays a crucial role in improving child health. Promotion of hygiene through provision of safe drinking water supply, water treatment, and improved sanitation has demonstrated 56% difference in the risk of acquiring diarrhea for children attending intervention vs. control schools in water-scarce sites in Kenya (adjusted risk ratio (aRR) 0.34, 95% CI 0.17–0.64) [15]. In Nigeria, majority (55.8%) of the school students were dissatisfied with the waste disposal mechanism at school [16]. A study conducted in Ghana indicated that school children despite being informed about the significance of handwashing were not able to practice due to lack of hygiene enabling facilities at the schools [17]. Thus, schools’ physical environment has a strong influence on children’s overall hygiene practices.

Various school-based interventional studies have shown improvement in enhancing personal hygiene among school children. A study in Nigeria showed significant improvement in primary students’ practice of keeping cleanliness after school-based health education on personal hygiene [18]. Likewise, KAP survey in India demonstrated improvement in school children’s personal hygiene after receiving health education program [19]. In addition, oral health education program in Bangladesh depicted significant improvement in school children (grade 6–8) KAP from baseline, alongside a reduction in dental cavities [20]. Furthermore, Kenya’s school water, sanitation, and hygiene (WASH) interventions documented reduction in diarrhea-related outcomes among children under 5 years of age [21].

Under school health education programs, ‘child to child’ approach has been widely used for improving health outcomes among children [22, 23]. In Kenya, children were educated to promote handwashing. School children built handwashing stations inside their homes and also persuaded their parents to build latrines [22]. Furthermore, school-based hygiene curriculum has also been used as a strategy to promote hygiene practices at school and at home settings among school children [24]. While school-based programs are important to promote child health, the role of mothers/ caretakers’ improved hygiene knowledge has also been documented to contribute in improved health outcomes for children [25].

In the context of Pakistan, alongside pneumonia and diarrhea, [14] worm infestation, [26] scabies, [27] and dental carries [28] among school age children are the commonly reported health issues manifesting poor personal hygiene. An evaluation study on WASH interventions on school children's hygiene behavior change in two cities of Pakistan demonstrated that almost 48% of the government school children avoid school toilets due to poor sanitary conditions [29]. In the local context, KAP surveys have been conducted assessing some of the components of hygiene; which does not provide a comprehensive hygiene assessment among younger children and also lacks the hygiene intervention component [28, 30, 31].

It is unfortunate that ‘school health’ remains a neglected aspect of public health in Pakistan [32]. In 2005, the School Health Program was launched in 17 districts of the country by the Ministry of Education, Pakistan in collaboration with United Nations Educational, Scientific and Cultural Organization (UNESCO). The program focused on health screening services for school children and ignored the health education component, however this program couldn’t sustain for long [33]. Gaps in school health promotion highlighting the need for hygiene awareness and intervention has been addressed in WASH programs across LMICs; with potential to contribute towards Sustainable Development Goals (SDGs) goal 3 [34, 35]. This further necessitates the need for implementing and evaluating school-based interventions to exhibit improvement in hygiene behavior among children.

The scope of this research is to determine the effectiveness of school-based intervention program; encompassing behavior change communication (BCC) strategies and bringing improvement in the school settings through streamlining improved drinking water facility and adequate garbage disposal (as per need), while addressing holistic aspect of hygiene. To the best of our knowledge, there is paucity of studies in Pakistan on school-based interventions to promote personal and environmental hygiene among school children. Towards designing school-based interventions, involvement of parents and teachers must be considered as they are important stakeholders for school going age children. In addition, they further have detrimental effects in shaping children’s overall health and hygiene behavior. Comprehensive assessment about personal and environmental hygiene factors among primary school children by involving teachers and parents has remained a gap in the local context, which this study attempts to address whilst testing a school-based hygiene intervention model.

In this paper, we therefore present a protocol for school-based intervention study using mixed-methods design to be implemented in schools of peri-urban community settings in Pakistan. The proposed study aims to improve the knowledge and practices of school children towards adaption of personal and environmental hygiene through school-based interventions. To the best of our knowledge, this research is first of its kind in the country involving caregivers (mothers), health, and education authorities in undertaking holistic hygiene assessment using mixed methods approach to improve hygiene behaviors among primary school children. Earlier studies in the local context have only assessed KAP of school children on selected hygiene components. In context of public sector schools in Pakistan, this research is an effort to demonstrate the effectiveness of school-based interventions using BCC tools to enhance hygiene awareness and practices among school children (using adult-to-child and child-to-child approach); an idea which has never been tested before in the local context. Furthermore, this research will also evaluate the integration of hygiene concepts into the education curriculum; which remains unique to this study. Moreover, this study will also attempt to unfold the perspectives of key stakeholders (teachers, mothers, health and education authorities’ representatives) to understand the factors contributing to the adaption of hygiene behaviors among children.

Primary research questions include;

1. Does school-based hygiene interventions facilitate improvement in knowledge and practices among primary school children studying in semi-urban schools in Karachi, Pakistan?
2. What are the enablers and barriers towards the adaption of personal and environmental hygiene practices by school children enrolled in semi-urban schools in Karachi, Pakistan?

Secondary research questions include;

1. Does mothers’ improved hygiene knowledge and practices enhance children hygiene knowledge and practices at semi-urban schools, in Karachi, Pakistan?
2. Does school-based hygiene interventions contribute in reducing the prevalence of communicable illnesses among primary school children at semi-urban schools in Karachi, Pakistan?

Specific research objectives of the study include;

1. Improve hygiene literacy and practices among primary school children by 10–15% (from pre-intervention to post-intervention) during October 2019 - December 2020 at semi-urban schools, Karachi, Pakistan.
2. Explore factors (facilitating and constraining) towards the adaption of personal and environmental hygiene practices among primary school children at semi- urban schools, Karachi, Pakistan during the pre-intervention phase by February 2020.
3. Determine the role of mothers with improved knowledge and practices in personal and environmental hygiene to enhance knowledge and practices of their children enrolled at semi-urban schools, Karachi, Pakistan over the period of January 2019- August 2020.
4. Estimate the overall change in the prevalence (increase or decrease) of communicable childhood illnesses among primary school children during October 2019–December 2020 (pre to post-intervention) in Karachi, Pakistan.

## Methods

The study will employ quasi-experimental design (pre-post-intervention without a control arm). The proposed study design is chosen as it will facilitate in evaluating school-based interventions in the selected school settings without randomization. Similar to the randomized trials, quasi-experiments (community-based trial) aims to demonstrate causality between an intervention and an outcome at the defined interval [36].

The study will be conducted in Gaddap town, Malir district, in Karachi, Pakistan. Gaddap town has 8 union councils with over 400 villages. Male members in the community mainly contribute to household income by working as laborers and farmers. A few are also involved in military and protective services. The vast majority of the married females are housewives, and few are employed in the health and education sectors. The majority of the dwellings in the community is composed of pucca (concrete) houses. Majority of the households utilize wood as cooking fuel. Most of the households fall under the lowest to middle wealth quintile. The catchment population avail health care services from the private sector due to unavailability of public sector health care facilities in the area. Community members mainly use boring as a source of water [37]. Sindh Education Textbook curriculum is followed in the schools. The government schools charge minimal fees. The cost of the textbooks and other educational expenses are to be borne by parents. Malir district has approximately 350 primary schools. Some of these schools are co-education, while the rest are gender-specific. Students’ enrollment varies from 70 to 150 per school in the locality (as per the information received from Taluka Education Office). Except for a non-governmental organization (NGO) adopted government school which offers health care services to children; with an embedded school health program, the rest of the schools in the area completely lack health services for children.

The study participants are (1) the primary school children, (2) mothers of school children, (3) teachers belonging to the primary section of school and (4) key informants, including Taluka Education Officer (TEO) and Taluka Health Officer (THO).

The sample size of the school children was determined using NCSS Pass version 16 software. To achieve 80% power for detecting a mean difference of 5.0 and a significance level (alpha) of 0.05 for a two sided paired *t*-test, and after adjusting for refusals and dropouts, it was rounded off to 277 pairs of participants. To achieve the desired sample of school children, census approach will be applied to recruit all children enrolled in the primary grade of the three schools. Same approach will be used to recruit the mothers of these sampled school children. The schools will be selected upon the recommendation of TEO to improve the hygiene situation in the sampled schools. Keeping in view the data obtained from the Taluka Education Office for an average enrollment of school children (70–150 children per school), three schools will be selected for the purpose of this study. One of the participating schools will be an NGO adopted school, and two government managed schools.

Inclusion criteria for children include students enrolled in primary grade (class 1–5) with informed consent given by mothers. After obtaining the mothers’ consent, assent will be obtained from the children at the respective schools. The school children will only be interviewed after obtaining their free will to participate in the study. If consent from child’s mother/ assent from child is not obtained, s/he will not be included in the study. Once the child gets enrolled into the study, child’s mother will be approached for her consent to participate. If the informed consent is not obtained from the mothers, she will not be able to participate in the study. For school teachers, those who are available at the time of the study will be recruited after their informed consent. On average, 2–3 teachers (per school) will be approached to participate in the selected schools. Teachers with unwilling attitude (during informed consent process) will not be included in the study.

In addition, we will also interview key informants; TEO and THO to explore their perceptions about enablers and barriers for the adaption of hygiene practices among primary grade school children. Interviews with these key respondents will be held after obtaining their informed consent.

Inclusion criteria for schools include its location in a semi-urban setting in Karachi, Pakistan and willingness of the school management in executing the research activities by the study team.

The study is built on mixed methods data collection approaches to gain insight of the hygiene literacy and practices among school children, teachers and mothers. Table 1 presents a snapshot of research participants, different data collection methods, its frequency, sampling strategy, data analysis plan in accordance with the research objectives and indicators.

**Table 1:** Summary of study objectives and methods

Serial Number	Objectives	Indicators	Study Population	Data collection methods	Frequency	Sampling strategy	Data analysis
1.	Improve hygiene literacy and practices among primary school children by 10-15% (from pre-intervention to post- intervention phase) during October 2019 – December 2020 at semi-urban schools, Karachi, Pakistan	▪ Improved hygiene knowledge among school children by 10-15% ▪ Improved hygiene practices among school children by 8-10%	School children enrolled in primary grade at the sampled schools	Survey using questionnaire	Baseline & Endline	Census	▪ Proportions and mean scores will be calculated ▪ Use of statistical tests: ◦ McNemar test ◦ Paired *t*-test
2.	Explore factors (facilitating and constraining) towards the adaption of personal and environmental hygiene practices among primary school children at semi-urban schools, Karachi, Pakistan during the pre-intervention phase by February 2020	▪ Factors facilitating hygiene behaviors among school children ▪ Factors restraining hygiene behaviors among school children	▪ Mothers of school children enrolled in primary grade at the sampled schools ▪ School teachers currently deployed at the primary grade in the sampled schools ▪ Taluka Education Officer ▪ Taluka Health Officer	Focus group discussion guide for mothers In-depth interview guide (specific for teachers and other respondents)	Baseline & Endline	Purposive	Thematic data analysis
3.	Determine the role of mothers with improved knowledge and practices in personal and environmental hygiene to enhance knowledge and practices of their children enrolled at semi-urban schools, Karachi, Pakistan over the period of January 2019- August 2020	▪ Improved hygiene knowledge among the mothers of school children by 10-15% ▪ Improved hygiene practices among the mothers of school children by 10-15%	Mothers of school children enrolled in primary grade at the sampled schools	Survey using questionnaire	Baseline & Endline	Census	▪ Proportions and mean scores will be calculated ▪ Use of statistical tests: ◦ McNemar test ◦ Paired *t*-test
4.	Estimate the overall change in the prevalence (increase or decrease) of communicable childhood illnesses among primary school children during October 2019- December 2020 (pre to post- intervention phase) in Karachi, Pakistan	▪ Percent change (increase of decrease) in the prevalence of communicable diseases among primary school children	Mothers of school children (recruited in the study) will be interviewed	Survey using questionnaire	Baseline & Endline	Census	▪ Proportions and mean scores will be calculated ▪ Use of statistical tests: ◦ McNemar test ◦ Paired *t*-test

Assessment of personal and environmental hygiene in our study is based on the aspects endorsed by World Health Organization (WHO) and United Nations International Children’s Emergency Fund (UNICEF) as a set of practices and conditions for better health maintenance and prevention of diseases. As defined by Boot and Cairncross (1993), hygiene is “the practice of keeping oneself and one’s surroundings clean, especially in order to prevent illnesses or the spread of diseases” [38].

Furthermore, a set of hygiene indicators assessed by earlier studies [1, 11, 12, 14, 16, 26, 39, 40] were also referred and incorporated in this study. Table 2 depicts the set of hygiene indicators which will be referred in this study.

**Table 2:** Operational definitions of hygiene indicators

Serial Number	Hygiene indicators	Operational definitions
1.	Personal hygiene	1. Drinking boiled/chlorinated/filtered water 2. Handwashing (pre and post meal, after defecation, and after playing) 3. Tooth brushing with toothpaste 4. Keeping nails short 5. Covering mouth with elbow while sneezing and coughing 6. Taking bath regularly (at least once daily) 7. Washing fruits and vegetables before eating
2.	Environmental hygiene	1. Not spitting on streets 2. Not throwing garbage/waste on streets 3. Maintaining cleanliness of school toilets

**Fig. 1:**
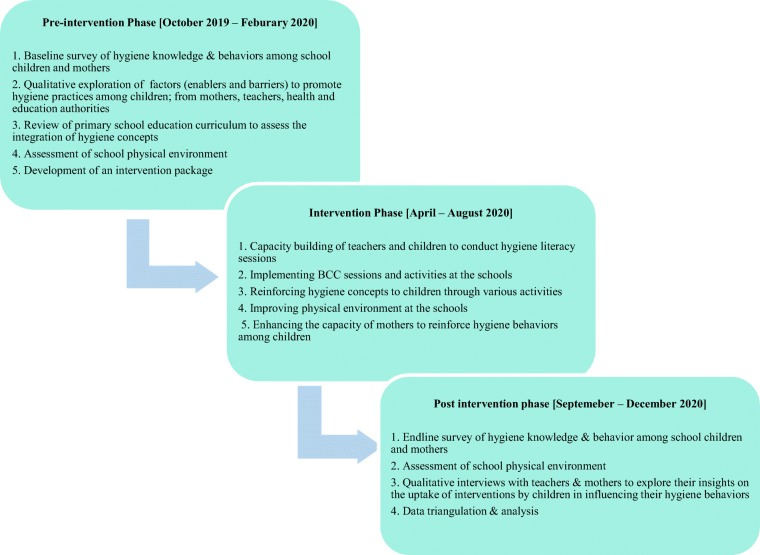
Project stages [Presentation of project stages alongside the time frames and key activities]

The study has been structured into three phases – pre-intervention, intervention and post-intervention with timelines and major activities depicted in Fig. 1, with details narrated in the below section.

### Phase I: Pre-intervention

Before data collection, community stakeholders (teachers and school management) will be taken on board and information about the overall scope and objectives of the study will be shared with them. Meetings with the community and the school leadership will remain a significant step to seek their cooperation throughout the study. All data collection tools will be translated into the local languages (Urdu and Sindhi). This phase will also involve pre-testing of all the data collection tools in the neighborhood school within the catchment area. After pre-testing, necessary modifications will be carried out to finalize the tools. Details of the mixed methods data collection approaches are mentioned in the following sections.

The quantitative data collection instruments include two closed-ended survey questionnaires (for students and mothers). Survey questionnaire for children include questions to gauge children’s knowledge and practices on basic personal and environmental health aspects at pre and post-intervention phases of the study. Aspects under personal and environmental hygiene are highlighted in Table 2. All children will be interviewed at the school settings.

On the other hand, survey questionnaire for mothers include questions about socio-demographic information (including age, qualification, occupation, income, household assets etc.). To obtain the demographic information, the Pakistan Demographic and Health Survey (PDHS 2012–2013) [41] survey tool was adapted. Followed by the demographic questions, mothers will be particularly inquired about their knowledge and practices related to personal and environmental hygiene. In addition, the questionnaire will also assess the hygiene habits of their child. In case of more than two children belonging to the same mother, she will be inquired about the hygiene habits of a child through random selection. Interviews with the mothers will be held at their homes. Furthermore, children’s health issues (with particular attention on infectious diseases) in last 1 month will also be inquired from the mothers. This will include some diagnostic questions to rule out the occurrence of diarrhea, acute respiratory infections (ARI), scabies, typhoid, malaria and worm infestations (commonly prevalent in the local context) among school children. A set of diagnostic questions were developed by referring to the Centers for Disease Control and Prevention (CDC) guidelines [42] and taking expert opinion from the Family Medicine Consultant at the Aga Khan University & Hospital, Karachi, Pakistan. For the diagnostic questions related to diarrhea and ARI, PDHS tool, 2012-2013 tool [41] was referred.

The role of schools in promoting hygiene behaviors among school children will be assessed by observing the physical environment. The observations will be categorized as present and absent with comments in six domains. This includes general maintenance, waste disposal, handwashing facility (including, water availability, water storage and availability of soap), sanitation facility (functionality, hygiene, and availability of cleaning agents), drinking water facility and hygiene behavior of school children. The school physical environmental assessment checklist has been adapted from the WHO WASH standards in low cost settings [43].

By using qualitative data collection approach, four tools will be used; (1) checklist to review the school education curriculum, (2) school hygiene assessment checklist, (3) in-depth interview guide with school teachers, TEO and THO and (4) a focus group discussion (FGD) interview guide for mothers.

School education curriculum checklist has been designed to assess the integration of basic health and hygiene aspects into the primary education curriculum (Sindh Text Book of class 1–5 grade). This includes the assessment about the integration of key concepts related to personal and environmental hygiene into the primary education curriculum, the use of strategies to increase hygiene literacy among children (for instance, pictorial messages or text only), whether or not the curriculum sensitize children about the unhygienic condition which leads to illnesses. Furthermore, while reviewing the curriculum, the structure of the language will also be examined for its simplicity and attractiveness for catching children’s attention. All the findings will be documented on MS Word with columns having the above components. The observation checklist has been adapted from the curriculum integration and instruction alignment guide by Washington state [44].

In-depth interviews (IDIs) will be carried out with the school teachers in the selected schools (associated with teaching in the primary section) and representatives in the Taluka Education and Health Office. The purpose of IDIs with these stakeholders is to explore their perceptions on hygiene literacy and practices among school children and to explore the factors which positively or negatively influences children’s hygiene behaviors. The interviews with TEO and THO will be meaningful to understand their role in health promoting activities at the schools and the collaboration (if any) exists between the two sectors. Purposive sampling technique will be employed to recruit school teachers from the sampled primary schools, mothers of children enrolled at the sampled schools and key informants (from Taluka Health and Education Authorities) at present. An estimated length of the IDI will be 30–40 min.

FGDs will be carried out with the mothers of children who are enrolled at the sampled schools. To facilitate interview process, a FGD guide has been developed. FGDs will be instrumental in exploring the mothers’ views on health and hygiene, their knowledge and practices about hygiene and also their children’s hygiene practices. In addition, FGD would also explore perceptions of mothers on innovative strategies to promote hygiene knowledge and practices among school children. Approximately, 2–3 FGDs with mothers will be carried out per school with approximately 8–10 participants in each FGD. The number of FGDs will be increased keeping in view data saturation. Setting for the FGD will be decided in consultation with the mothers.

The IDIs with teachers and other stakeholders and FGDs with mothers will be pivotal in exploring their views on ‘what can and cannot work towards enhancing personal and environmental hygiene’ among school children. The perceptions and opinions gathered during the FGDs will be meaningful in modifying the intervention package.

Separate interview guides for all key respondents will be developed with probes to facilitate the interviews process. Information on the survey form will be filled by data collectors, whereas IDIs and FGDs will be conducted by the Principal Investigator (PI) and co-investigators.

### Phase II: Intervention

**Fig. 2:**
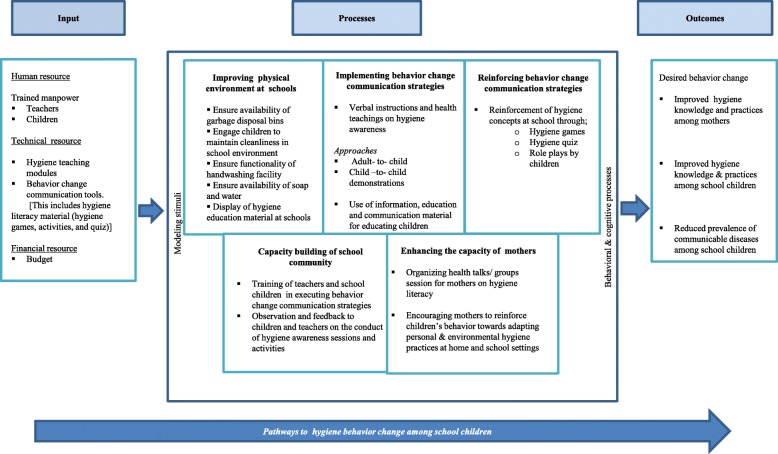
Conceptual Framework: Pathways to hygiene behavior change among school children [An illustration of input, processes and outcomes to hygiene behavior change among school children]

The multi-component intervention package will be designed aiming to improve hygiene literacy and behaviors among children. This includes improvement in the school physical environment, implementation of BCC strategies, reinforcement of BCC strategies, capacity building of the school community and enhancing the capacity of mothers to facilitate school children adapt hygiene behaviors. The details of the activities under each component are highlighted in Table 3, with pathways to hygiene behavior change among school children illustrated in Fig. 2.

**Table 3:** Intervention package to improve hygiene literacy and practices among primary grade school children & their mothers

Serial Number	Interventions	Frequency	Use of strategies
1.	Capacity building of school community (master trainers)		
	1.1 Teachers’ training/refresher sessions will be arranged to enhance hands on skills for educating children about improving personal and environmental hygiene	Once during the intervention phase and refresher sessions will be arranged as per need	Training sessions per school by using audio and visual aids
	1.2 Pool of children with good leadership skills will be selected from the schools and will be trained in educating others on personal and environmental hygiene concepts	One time activity of selection of children	Classroom observations to identify children with good leadership skills
2.	Implementing behavior change communication strategies		
	2.1 Children will be educated about the need for hygiene (personal and environmental hygiene). The sessions will be organized by teachers	Thrice in the entire duration	Use of posters and graphics in the local language
	2.2 Awareness raising sessions for mothers will be conducted by teachers and health workers to enhance mothers’ practices for personal and environmental hygiene	Once every 2 weeks for 4 weeks	Pictorial presentations in the local language
3.	Reinforcing behavior change communication strategies		
	3.1 Reinforcement of hygiene concepts to children through multiple strategies	Once every 2 weeks	Hygiene diary, hygiene games, and hygiene quiz
	3.2 Role plays and awareness raising sessions by the children (senior students) to promote hygiene among school children	Once every 2 weeks	Role plays and theatre
4.	Improving physical environment at schools		
	4.1 Environment will be made conducive through audio visual aids to foster adaption of hygiene habits among children	To be displayed at schools during the entire intervention phase	Cartoon characters demonstrating hygiene habits, and display of posters with hygiene messages
	4.2 Improvement in school physical environment through ensuring the availability of garbage disposal bins, soap and water, functionality of handwashing facility, and engaging children to maintain cleanliness in school environment Note: Improvement in the physical environment of the selected schools will be undertaken in close collaboration with the district/ taluka education authorities and a local NGO	To be carried out and ensured in the entire intervention period	Dissemination of the findings from the pre-intervention phase to schools’ administration
5	Enhancing the capacity of mothers		
	5.1. Mothers of school children will be sensitized on hygiene aspects through group discussions	Thrice in the entire period	Health education flyers and posters to be used during the group discussion sessions at home/ school settings

**Fig. 3:**
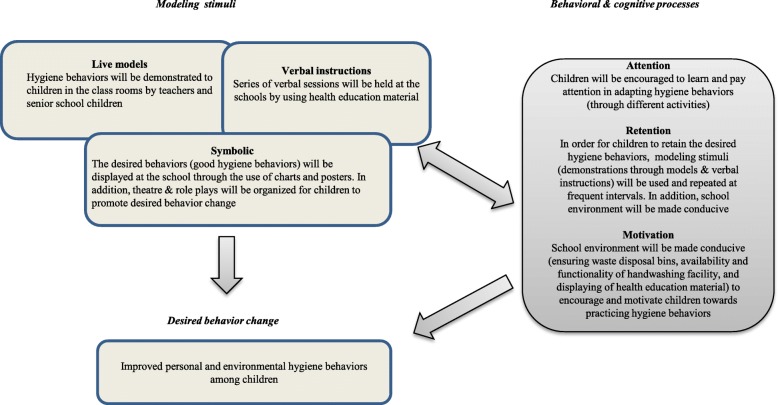
Application of Albert Bandura’s Social Learning Theory to promote hygiene behavior among school children. [The components in the Albert Bandura’s Social Learning Theory has been reflected for its use in promoting hygiene behavior among school children in the proposed study]

The entire intervention phase (expected to last for 4–5 months) has been conceptualized by utilizing the Albert Bandura’s Social Learning Theory [45]. The theory postulates that learning takes place within a social context with three different modeling stimuli (live models, verbal instructions and symbolic). Hygiene behaviors will be modeled by teachers and senior school students at the schools. By using health education material, series of health sessions will be conducted. The school environment will be made symbolic to learn and practice hygiene behaviors through display of information, education and communication (IEC) material and through organizing drawing and writing competition for children on hygiene themes at the school settings. In addition, various behavioral and cognitive processes will be given attention. This includes encouraging children to learn and pay attention to the hygiene behaviors through different teaching and learning strategies. Retention of key concepts will be fostered by repeating the sessions at the frequent intervals. To motivate the children to practice hygiene behaviors, school environment will be made conducive by ensuring functional handwashing facility with soap, improve drinking water facility and availability of garbage disposal bins. These infrastructural changes, alongside BCC strategies will motivate children to practice and demonstrate the learned behaviors through improved hygiene literacy. Refer Fig. 3 for the application of Albert Bandura’s Social Learning Theory in the study.

During the entire intervention phase, the school leadership will be taken on the board to plan and execute the activities. Alongside ‘adult-to-child’ approach, notion of ‘child-to-child’ approaches [21, 22] in BCC will also be embedded in the intervention. Field manual for the intervention modules will be developed to ensure adherence of the field team with the proposed interventions. The intervention will be administered by the PI and field team who will be thoroughly trained in using the intervention modalities. Refer Table 3 for the detailed intervention package.

Note: Although improvement in sanitation facilities is essential to promote hygiene practices, however keeping in view the budgetary constraints in the study, sanitation facility cannot be upgraded. An attempt will, however, be made to initiate dialogues with Taluka and District Education Authorities and school administration to sensitize them about the need for improved sanitation facility at the schools.

### Phase III: Post-intervention

Post-intervention phase will determine the effectiveness of the school-based interventions by measuring the level of change in the hygiene literacy and practices of school children and mothers through survey questionnaires (as executed in Phase I).

Perceptions of mothers and teachers will also be gathered on how well the intervention modalities worked at the respective school settings and will explore respondents’ perceptions of the interventions in influencing children’s hygiene knowledge and practices. Inspection of school physical environment will also be carried out to ascertain improvements in the school environment using the physical environment assessment checklist.

Across all phases of the study, PI and co-investigators will randomly visit the field sites to ensure monitoring of data collection and implementation of the intervention modalities. Feedback will be shared with the field team to ensure strict adherence to the data collection steps and intervention aspects. Data collection forms, interview recordings, and transcripts will be safely stored with the PI, while providing its access to the research team for data analysis.

Analysis will be carried out for pre-post intervention phases for the data obtained at the child level (hygiene knowledge and practices), mothers’ level (hygiene knowledge and practices for mothers and for their children). This phase will also include physical environment assessment at the schools and exploration of stakeholders' (mothers, teachers, health and education authorities' representatives) views on factors influencing hygiene behaviors among school children. Refer Table 1 for the data collection methods.

Quantitative data derived from the survey questionnaires (for children and mothers) will be entered in EpiData version 3 and will be analyzed in SPSS 19.0. Proportions will be reported to present knowledge and practices for children and mothers’ personal and environmental hygiene. Proportions will be categorized into knowledge and practices domains under personal and environmental hygiene. Mean proportions will be then converted into scores (< 50 = poor, 50–75 = good and > 75 = excellent). The scoring criterion has been customized for the purpose of analysis.

The McNemar test will be used to analyze the differences in the proportions for knowledge and practices of school children pre and post-intervention. Similar test will also be applied to analyze the differences between the proportions of knowledge and practices of children and their mothers; before and after the intervention. Paired *t*-test analysis will also be applied pre and post-intervention to measure the differences in knowledge and practice scores between mothers’ hygiene literacy and practices with their children’s knowledge and practices. Besides this, similar test be applied to assess the differences in children’s hygiene knowledge and practice scores pre and post-intervention. (< 50 = poor, 50–75 = good and > 75 = excellent). In addition, mean proportions will be calculated to measure the prevalence of communicable diseases among school children for pre-post intervention phases.

Qualitative data including FGDs (with mothers) and IDIs (with school teachers, TEO and THO) will be recorded and transcribed verbatim. Textual data will be manually analyzed. Thematic analysis will be carried out in accordance with the steps described by Graneheim & Lundman, 2004 [46]. Meaning units (recorded text) will be read several times to identify the codes (short meaningful descriptions). Similar codes will be then clustered into groups called “categories”, which will be later classified into themes. Themes generating from the data set will represent the latent content (the underlying meaning of the text) and relationships among the categories.

In addition, review of primary school education curriculum will be carried out keeping in view the personal and environmental hygiene components operationalized for the study (refer Table 2).

Data obtained from the qualitative and quantitative tools will be triangulated for analysis at the end of the post-intervention phase. In order to obtain a comprehensive understanding of hygiene literacy and practices among children and whether or not the school-based interventions have positively improved knowledge and practices of children, information from the multiple sources (surveys, FGDs, IDIs, school physical environment assessment and education curriculum review) will be triangulated.

## Discussion

Communicable diseases among school children remain a highly prevalent issue across LMICs [47, 48]. School and home are two primary settings to plan and implement BCC on hygiene [49, 50]. The described protocol of the study aim to test the effectiveness of school-based hygiene interventions to improve the knowledge and practices of school children in semi-urban schools in Pakistan. Refer Fig. 2. The intervention will be conducted at three schools (one NGO-adopted and two government-managed schools) in urban squatter settlement with multiple stakeholders’ involvement (school children, mothers, Taluka Health and Education Authorities).

Maternal knowledge and practices, as well as those of school teachers, have shown to play an imperative role in improving hygiene practices of school children [51, 52]. During the pre-intervention phase, alongside survey, interviews will be held with the key stakeholders to seek their perspectives on children’s overall hygiene literacy and practices. Stakeholders’ opinions regarding positive and negative influences on children’s hygiene practices at school and home will also be sought. Additionally, the school physical environment will be observed to inspect the availability of soap and appropriate handwashing facilities, general cleanliness, adequate garbage disposal facilities, etc. for the students and staff at the school premises. During the intervention phase, participatory sessions will be organized with the school teachers to obtain their feedback on the intervention package. In the later stage, teacher’s capacity building sessions will be carried out to help them facilitate hygiene awareness sessions at the schools. In addition, behavior change sessions for mothers will also be conducted at their homes. The study also attempts to analyze the existing school education curriculum and examine the extent to which it incorporates hygiene principles. Furthermore, based on the observations of schools’ physical environment in the pre-intervention phase, improvement in the handwashing facility and garbage disposal mechanism will be carried out in the identified school settings.

The effectiveness of the interventions will be gauged through an endline survey of children and mothers’ hygiene knowledge and practices, and also by capturing mothers and teachers’ perceptions on the effectiveness of school-based interventions on the overall hygiene literacy and practices of children. In addition, study would also enable us to measure the change (if any) in reducing the prevalence of communicable diseases among children from the baseline.

The use of multiple data collection tools will facilitate us to validate our findings and assumptions about the children’s hygiene knowledge and practices. Principles of Albert Bandura’s Social Learning Theory are incorporated into the study’s intervention phase to reinforce children’s hygiene practices through use of different modeling stimuli (live modeling, symbolic and verbal instructions) and through different behavioral and cognitive processes which can potentially influence the adaption of hygiene behaviors among children. Adequate knowledge about hygiene practices will be reinforced at frequent intervals at the study settings.

To the best of our knowledge, earlier studies aimed at improving hygiene literacy and practices at schools have not taken the holistic approach as conceptualized in this research protocol. This study, therefore, intends to undertake a comprehensive assessment of hygiene by not only assessing children’s knowledge and practices, but also attempts to unfold the enablers and barriers of hygiene knowledge and practices for school children by involving different stakeholders. And by undertaking a comprehensive assessment of school physical environment and education curriculum. As this is a school-based interventional study aiming to improve the knowledge and practices of children on basic hygiene; whether or not children demonstrate hygiene behaviors while playing or at home is beyond the scope of this study. Hence, it’s not practically possible to be vigilant of their practices and behaviors round the clock. Mothers’ post-intervention survey will serve as a proxy to assess children’s overall approach in maintaining hygiene practices at home and while playing. Although, health literacy sessions will be organized for the mothers of school children, however this may or may not result in positive behavior change among children due to the continuation of poor hygiene habits at home setting and lack of positive reinforcement by parents. This may pose a limitation towards positive change in hygiene practices among children, even after multi-pronged intervention. Some of the methodological limitations related to the chosen study design include an absence of a control group, lack of sustained behavior change measurement among children after an endline assessment, possible drop out of few children from the schools and events occurring concurrently with the intervention may contribute to the observed and reported behavior change among children [53].

The study findings would be would be useful in recommending the practice of hygiene BCC sessions at the school as part of the primary education curriculum, modifications into the primary education curriculum to incorporate hygiene concepts, and ways to improve the schools’ physical environment to enable children practice hygiene behaviors. Moreover, research and policy brief would be developed to initiate dialogues with the Taluka and District Education Authorities to seek their attention towards improving hygiene practices among children and also to improve physical environment at the schools.

Quality assurance practices during the conduct of the research will remain a cornerstone of the project. PI will supervise and closely monitor all the activities. Project management activities are described in the following section encompassing all three phases. Finance Department at CHS, AKU will facilitate in the costing and budgetary matters.

During Phase I, PI will recruit the data collection team members including field coordinator and data collectors. Their training will be conducted by PI and co-investigators. While training the field team, PI in consultation with the community stakeholders and Taluka Education Authority will identify the schools for the purpose of the study. Data collectors will be delegated the responsibility to pre-test study tools under the supervision of field coordinator. In addition, the study team will also supervise the baseline data collection. In the later stage, data entry (for quantitative data) will be managed by data entry personnel and qualitative data will be manually coded by the study team.

The Phase II will involve consultative meetings between the study team, field coordinator and school teachers to plan and implement the proposed intervention package. During meetings, stakeholders’ suggestions will be incorporated and findings obtained during the pre-intervention phase will also be considered in modifying the intervention package. Later, to implement the intervention (as proposed in Table 3 and in accordance with Albert Bandura’s Social Learning Theory), PI will train local community workers (as data collectors), some school children and teachers. Field coordinators and study team will conduct supervisory visits to the field sites to ensure adherence to the proposed activities.

Following Phase II, PI in collaboration with the study team will ensure post-intervention data collection at the field sites. Quantitative data will be entered in Epi Info and EpiData software and will be analyzed by the co-investigator trained in Epidemiology and Biostatistics. On the other hand, qualitative data will be manually coded by PI and the study team. Following this, data will be triangulated. Confidentiality of the data and privacy of study participants will be ensured throughout the study by the research team.

## Data Availability

The data collection tools developed/ adapted in the study and all data sets will be available upon request. Please note that there are no formal publicly available repositories in Pakistan for research manuscripts. PI can be contacted to access data files.

## Abbreviations

AKU: Aga Khan University
ARI: Acute Respiratory Infection
BCC: Behavior Change Communication
CDC: Centers for Disease Control and Prevention
CHS: Community Health Sciences
ERC: Ethics Review Committee
FGDs: Focus Group Discussions
IDIs: In-depth interviews
IEC: Information, Education and Communication
KAP: Knowledge, attitude and practices
LMICs: Low-middle income countries
NGO: Non-governmental Organization
PDHS: Pakistan Demographic and Health Survey
PI: Principal Investigator
REPDS: Rural Educational Promotion and Development Society
SDGs: Sustainable Development Goals
TEO: Taluka Education Officer
THO: Taluka Health Officer
UESCO: United Nations Educational, Scientific and Cultural Organization
UNICEF: United Nations International Children’s Emergency Fund
USAID: United States Agency for International Development
WHO: World Health Organization
